# Application of Intelligent Intravenous Drug Dispensing Robot in Clinical Nursing

**DOI:** 10.1155/2022/4769883

**Published:** 2022-03-21

**Authors:** Lifen Zhang, Wei Liu, Yanhong Zhang

**Affiliations:** ^1^Pharmacy Intravenous Admixture Service Center, Harbin Medical University Cancer Hospital, Heilongjiang, Haerbin 150000, China; ^2^PET/CT-MR Center, Harbin Medical University Cancer Hospital, Heilongjiang, Haerbin 150000, China; ^3^ICU Unit 1, Harbin Medical University Cancer Hospital, Heilongjiang, Haerbin 150000, China

## Abstract

In order to explore the application of intelligent intravenous drug dispensing robot in clinical nursing, the efficiency, residual amount, needle pushing, and pulling times, the incidence of accidental hand stab injury and the accuracy of drug dispensing were compared and observed between intelligent intravenous drug dispensing robot and manual dispensing. The study found that the sterile powder residual amount of injection is pointed out in the standard, the drug label is not equipped with ≤50 mg, and the residual limit is ≤ 15%; the lamination is 50–150 mg, the residual amount is limited to ≤10%; the marking is 150–500 mg, the residual amount is ≤ 7%; the marking is 500 mg, the residual amount is limited to ≤5%; 6 months of artificial deployment accidental stab ergonomics occur; without this phenomenon when using the drug-like robot, 6 months internal use of the pharmaceutical robot, 4 times of error occurrence, and errors occurred 60 times. During the clinical intravenous infusion dispensing process, the use of intelligent vein is used to configure the robot, improve the efficiency of the drug, reduce the incidence of drug residual, reduce the incidence of dispensing, increase the accuracy and safety of venous liquidation, and decrease to some extent professional injury caused by drug formulation for liquid nurses.

## 1. Introduction

In recent years, the intravenous drugs allocation center (pharmacy intravenous admixture services, PIVAS) development is rapid and has become the rational use of drugs as the core of the important content of pharmaceutical care. However, in the process of drug formulation, there are also a series of hidden dangers such as splashing of liquid medicine and leakage of aerosol, which are harmful to the health of the preparation personnel and drug pollution, air pollution, and environmental pollution; therefore, it is a new trend of hospital clinical development to actively carry out advanced intravenous drug dispensing technology [1]. Artificial intelligence is a cutting-edge technology in the current development of science and technology; it is widely used in the medical field in various forms, such as clinical medical diagnosis, surgery, neural network technology, expert system, and medical image diagnosis. In addition, it has also been applied in home care for the elderly [2]. In the dispensing work, different devices work together to realize the automation of dispensing process in the pharmacy, and the automatic configuration of intravenous infusion is an integral part of the automated dispensing in hospital pharmacy [3]. At present, most hospitals in China have intravenous infusion preparation in intravenous drug preparation centers and wards, and problems such as difficult dispensing process, high labor intensity, occupational injury, time-consuming, and laborious, and shortage of personnel are prevalent; it has significantly reduced the quality and efficiency of intravenous infusion preparation and is easy to cause nurseries and patients' disputes, which attracted great clinical attention [4]. Active development of advanced intravenous drug dispensing technology and innovative management mode is a new trend in the development of hospital pharmacy [5].

Studies have shown that the implementation of information technology affects the quality of patient care. Drug intravenous infusion is one of the important contents of the hospital clinical drug treatment, which is greatly related to the drug safety of patients. Therefore, there is an urgent clinical need for an automatic and intelligent solution dispensing equipment that can solve the above difficulties. Intravenous drug dispensing robot is a product of modern information technology and medical technology, and its application effect and safety in clinical intravenous infusion dispensing are currently highly concerned in clinical practice [6, 7]. After early practice, the dispensing robot is mainly used for dispensing sodium carlosulfonate, brain protein hydrolysate, complex coenzyme, cefazoxime sodium for injection, *Salvia miltiorrhiza* polyphenol, bone peptide for injection, and the withdrawal of predissolved cefathiamidine and piperacillin. The dosage of four kinds of drugs, carlosulfonate sodium, cefazoxime sodium for injection, complex coenzyme, and cefathiamidine, was relatively large in PIVAS of the hospital; different types and different counts are used (sodium carbosulfonate and complex coenzyme are common drugs, usually 4 and 2 capsules/bag, cefazoxime sodium for injection is an antibacterial powder, and predissolved cefathiamidine is equivalent to a water injection). The application of configuration robot in intravenous infusion drug preparation significantly improves the quality and efficiency of drug preparation, reduces the workload and psychological pressure of nursing staff, urges it to invest more time in the care of patients, improves the relationship between protecting patients to a certain extent, and improves the nursing service image of the hospital. Mukherjee U. and others proposed a dual goal: to maximize clinical outcomes and reduce the overall cost of robotic surgery in hospitals. The central robot in this research is the Da Vinci Surgical System. The background of the study is hysterectomy. We propose a comprehensive methodology that combines empirical analysis involving fields, forward-looking hospital surveys, retrospective analysis of hospital archive data, and modeling for robust examination and analysis, discrete event simulation identifies, and analyzes hospital policies that can be implemented. We analyzed specific policies related to the importance of robotic surgery in the light of the patient's disease status, the optimal size of the surgeon pool to facilitate the development of surgeon experience and learning, and the minimum level of experience of surgeons required to include the execution of robotic operations in the surgeon pool. The main contributions are as follows. First, hospital-level policies can help achieve clinical outcomes and cost-effectiveness for surgical robots [8].

Based on this, this study studied four representative drugs, namely, sodium carlosulfonate, cefuroxime sodium for injection, complex coenzyme, and cefathiamidine. By studying the cost-effectiveness and residue of these four drugs, the number of accidental stabbies and errors and the number of artificial assays are compared to artificial array, and the equipment is better than manualization. Pharmaceutical robots can significantly reduce the labor intensity of staff and have a good application prospect in the venous drug distribution center. More need to be described; the dispensing robot has also been further optimized while reducing the labor intensity of the staff, and the optimization of working mode has also been further optimized.

## 2. Research Methods

### 2.1. Materials

Sodium carlosulfonate, cefazoxime sodium for injection, complex coenzyme, and cefathiamidine were used in this study.

### 2.2. The Instrument

Pyjqr-f02a power injection dispenser was used.

### 2.3. Workflow of Dispensing Robot

**Figure 1:**
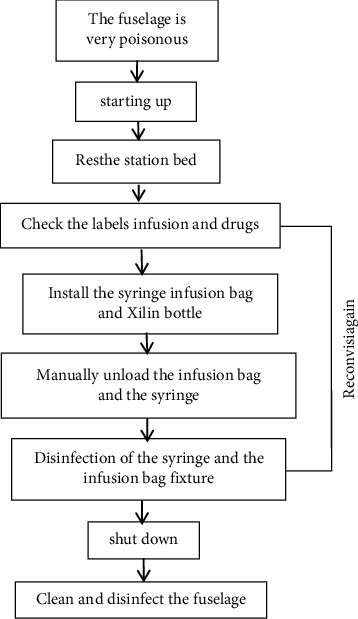
Workflow of dispensing robot.

The workflow of the dispensing robot from body disinfection to body cleaning and disinfection is shown in Figure 1.

### 2.4. Observation Indicators

1. Influence of dispensing robot on dispensing work efficiency
2. 10 PIVAS workers with different working years were randomly selected; one dispensing robot and one manual dispensing method were used to prepare four kinds of drugs, namely, carlosulfonate, cefazoxime sodium for injection, complex coenzyme, and cefathiamidine; the number of finished infusion bags allocated in the first and second hours of these two deployment methods was recorded, respectively, and the number of dispensing bags in one hour when one staff operated two dispensing robots at the same time to investigate the influence of dispensing robots on work efficiency.
3. Influence of dispensing robot on drug residues
4. According to the method under “1.4.1”, the empty bottles of medicine prepared in the two blending methods were kept, and the residual amount (residual volume) of each empty bottle was sampled according to the statistical method and recorded, respectively, and the average value was calculated.
5. Effect of the drug dispensing robot on the labor intensity of the staff
6. Taking four drugs of artificial deployment of sodium carlosulfonate, sodium cefazoxime for injection, compound coenzyme, and cefathiamidine, the number of flat push and pull needle cylinder within 1 h was calculated to investigate the impact of the drug dispensing robot on the labor intensity of the staff.
7. Effect of drug dispensing robot on the incidence of accidental stabbing in staff
8. Observe and record the number of accidental stabbing by the dispensing robot and manual deployment within 6 months.
9. Effect of drug dispensing robot on deployment accuracy
10. Observe and record the number of errors between the dispensing robot and the manual deployment within 6 months, so as to investigate the impact of the dispensing robot on the accuracy.

### 2.5. Statistical Methods

SPSS 22.0 statistical software was used for data entry and statistical analysis, $\bar{x}\pm s$ was used to describe the data, and the *t*-test or nonparametric test was used after the normal distribution was investigated. *P* < 0.05 was considered statistically significant.

## 3. Result Analysis

### 3.1. Comparison of Work Efficiency between Dispensing Robot and Manual Dispensing

The analyzed data conform to normal distribution, and the *t*-test is used. The results are given in Tables 1 and 2. As can be seen from Table 1, in the first hour of dispensing, the same staff dispenses the same medicine, and the manual dispensing speed is much faster than the robot dispensing. It takes time to install and replace syringes, fix infusion bags and vials when operating the dispensing robot, and the waiting time for dispensing is long. After a long time of manual deployment, people are prone to fatigue, and work efficiency will be reduced with the extension of time. From the second hour, the efficiency of manual dispensing has no obvious advantage over that of the dispensing robot; in the third hour, the working efficiency of the dispensing robot is higher than that of manual dispensing; in addition, the working efficiency of the dispensing robot is relatively stable within 3 h, which is not affected by the dispensing time. When one worker only operates one dispensing robot, the waiting time for dispensing is long; therefore, in actual work, one person can operate two machines at the same time; as can be seen from Table 2, within the first hour of dosing, the efficiency of operating two dispensing robots at the same time was significantly higher than that of manual dispensing.

### 3.2. Comparison of Residual Quantity between Dispensing Robot and Manual Dispensing

The analyzed data conform to normal distribution, and the *t*-test is used; the results are given in Tables 3 and 4. In the standard of sterile powder residue limit for injection, it is pointed out that the drug labeling load is less than or equal to 50 mg, and the residual limit is less than or equal to 15%; label 50–150 mg, residual limit ≤10%. For labeled loading of 150–500 mg, the residual limit is less than 7%, labeled loading >500 mg, residual limit ≤5%. The internal control standard of PIVAS in a certain hospital is 5% of the residual volume of solvent volume. When dispensing drugs, the dispensing robot generally extracts 1/3 of the vial volume as the solvent volume. The residual amount of 4 kinds of drugs prepared by the dispensing robot was tested, and the sample of each empty bottle and the average residual amount were qualified. As can be seen from Table 3, there was no significant difference in the average residual data of the same drug when 10 staff operated the dispensing robot, which indicated that different staff operating the machine had little influence on the drug residue; the residual amount of the same drug is relatively fixed and has no relationship with personnel and the number of operating machines [9]. The residual amount of artificial deployment of 10 workers have different data, which is related to each person's habit of adding medicine, the operation method. According to the analysis of data in Table 4, it can be seen that the three drugs of sodium carlosulfonate, cefazoxime sodium for injection, and complex coenzyme, the residual amount of medicine prepared by the dispensing robot is larger than that of manual dispensing. The reason is that the syringe of the dispensing robot is fixed vertically, for example, when there is no gap in the rubber plug of cefazoxime sodium for injection at the mouth of some cillin bottles, the liquid cannot be completely drained, and in manual blending, the liquid can be drained by inserting the needle tip in an oblique way [10]. In addition, the residual amount of cefathiamidine prepared by the dispensing robot was not significantly different from that prepared by manual dispensing, with no statistical significance; this may be related to the volume of the solvent of the dissolved drugs (2-3 mL of the three drugs, sodium carlosulfonate, cefazoxime sodium for injection, and complex coenzyme, is generally extracted for dissolution, and 5 mL of cefathiamidine is extracted for dissolution).

### 3.3. Manually Adjust the times of Pushing and Pulling the Needle

Taking four kinds of drugs, including manually mixed sodium carlosulfonate, cefazoxime sodium for injection, complex coenzyme, and cefathiamidine, as examples; the average needle push and pull times within 1 h were calculated, and the results are given in Table 5. According to the data in Table 5, the staff pushed and pulled the syringes for hundreds of times on average within 1 h; while the average daily allocation time of PIVAS in a hospital was 4-5 h, long-term repetitive movements with high frequency and intensity are easy to cause strain and deformation of the hand joints [11, 12]. The introduction of dispensing robot has solved this problem well; the staff only need to install the syringe and fix the sterilized infusion bag and vial on the machine; it reduces the operation of pushing and pulling the needle by the hand and greatly reduces the labor intensity of the staff.

### 3.4. Comparison of Accidental Stab Injury Rate between Dispensing Robot and Manual Dispensing

In 6 months, there were 6 times of accidental stab wounds on the hands in manual dispensing, while there were no accidental stab injuries on the hands of staff in the dispensing robot. As can be seen from the statistical data, due to improper or unskilled operation during manual deployment, the staff suffered 6 accidental stab wounds in half a year, while no such phenomenon occurred when the dispensing robot was used [13]. This is because when operating the dispensing robot, the staff only need to install the syringe, infusion bag, and medicine; during this process, the cap is worn on the tip of the needle until the robot starts mixing, and then, the cap is removed; at this time, the whole process is operated and deployed by the robot, and the staff will no longer touch the syringe and medicine, which greatly reduces the probability of staff being injured by friendly fire.

### 3.5. Comparison of Dispensing Accuracy between Dispensing Robot and Manual

**Figure 2:**
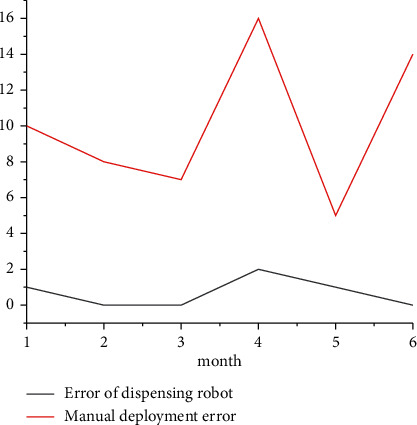
Comparison of dispensing errors between dispensing robot and manual.

The times of dispensing errors in PIVAS using dispensing robot and manual dispensing within 6 months were recorded, respectively, and the results are shown in Figure 2. As can be seen from the data in Table 6, in the statistical half a year, there were 10 cases of errors on average every month in manual dispensing, mainly in the three types of dispensing errors, dosage errors, and nonchanging syringes; however, the number of errors occurred in the use of the dispensing robot is relatively small, which is because the dispensing robot has selected the type and number of drugs on the setting page before dispensing; therefore, the probability of dispensing and dosing errors in the dispensing process is reduced; in addition, when 6 bags of infusion are deployed, the page will prompt to change the syringe; it will also prompt you to unload the syringe when changing the dosage, so that you will not make the mistake of mixing one medicine before changing the syringe. Several errors occurred by the dispensing robot were mainly due to the wrong number of setting drugs and the failure of the vial itself when applying medicine, which led to the reported loss of finished infusion.

## 4. Discussion

### 4.1. Advantages of Dispensing Robot

In the process of manual blending, syringes are used to add drugs, especially for multiple drugs, which requires repeated puncture of the bottle stopper; the tip of the needle may accidentally touch gloves, infusion bags, and surfaces, resulting in pollution; when the dispensing robot is used to install the vial and infusion bag, the cap is worn on the tip of the needle and is not removed until the deployment begins; in contrast to the fully closed equipment mentioned, the infusion bag and syringe need to be manually unloaded; however, the hand will not touch the feeding mouth and needle tip of the infusion bag in the blending process; when unloading manually, the needle cap will be automatically put on, and microbial pollution is greatly reduced. In addition, in the process of manual deployment, the staff is also easy to be accidentally injured by the tip, which also reduces some personal injuries [14–16]. In addition, when too much liquid (more than 10 mL) is extracted at one time during manual operation, it is easy to touch the pin of the syringe by the hand; the dispensing robot directly pushes and pulls the needle plug to extract 15 mL of solvent dissolved drugs each time, can extract up to 17 mL of liquid medicine at a time, and not only will it reduce contamination but it will also reduce the number of times you have to puncture the infusion bag. The dispensing robot can reduce the operation of pushing and pulling the needle by the hand, thus reducing the labor intensity of the staff. In actual work, the staff have certain strain and tenosynovitis in their hands due to long-term dosing work [17]. The dispensing robot can reduce the probability of staff being accidentally stabbed, reduce the dispensing error, and improve the accuracy of the work. In terms of work efficiency, the dispensing robot has obvious advantages in long-term dispensing; if it can be applied in a large number of PIVAS in the later period, one worker can operate two machines at the same time and thus can reduce the proportion of personnel, greatly reduce the labor intensity and occupational injury of staff, and reduce the workload and psychological pressure of nursing staff; it makes them have more time to invest in patient care, improves the nursing-patient relationship to a certain extent, and enhances the image of hospital nursing service.

### 4.2. Comparison between the Dispensing Robot and Similar Equipment

Now, there is also an automatic dispensing robot widely used in PIVAS; this automatic dispensing robot is a fully closed internal negative pressure device, large volume, simple dissolution mode, only shaking a movement, and the average working efficiency of each person per hour (42.13 ± 6.83) bag, mainly used for the deployment of antitumor drugs and antibacterial drugs. The dispensing robot in this research is small in size and can be directly placed in the biosafety cabinet or horizontal laminar flow table, manual suction action can be simulated, and there are a variety of dissolution modes; in addition to the deployment of antitumor drugs and antibacterial drugs, it can also be used for the deployment of various general drugs [18–20]. In terms of work efficiency, the literature only records the average number of bags allocated by the automatic dispensing robot per hour, and the specific drug varieties are not specified. From the perspective of the average number of dispensing bags, the dispensing robot researched in this study has certain advantages in terms of work efficiency. In addition, for the same equipment recorded in the existing literature, only the working principle, use process, and parameter setting optimization of the dispensing robot are introduced; there is no specific data analysis to reflect the advantages of the dispensing robot, but the data in this study reflect the advantages of the dispensing robot from five aspects.

## 5. Conclusions

This study found that the clinical application of intelligent intravenous drug configuration robot makes the operation of intravenous infusion more efficient, convenient, and safe, improve the efficiency of dispensing, and reduce the occurrence of drug residues and dispensing errors. In the process of drug solution allocation, people and drugs are completely isolated, providing effective protection for operators and reducing occupational injuries caused by drug allocation to nurses.

## Figures and Tables

**Table 1:** Comparison of work efficiency between one dispensing robot operated by one person and manual dispensing ($\bar{x}\pm s$).

Drug	1 hour		2 hours		3 hours	
	The machine efficiency	Artificial efficiency	The machine efficiency	Artificial efficiency	The machine efficiency	Artificial efficiency
Card collaterals sulfonic sodium	58.3 ± 8.6	83.4 ± 14.3	59.6 ± 9.2	65.6 ± 12.9	56.8 ± 7.5	53.7 ± 11.6
Cefazoxime sodium for injection	72.4 ± 12.2	91.2 ± 16.1	73.6 ± 10.4	76.2 ± 15.2	71.2 ± 9.7	64.9 ± 15.8
Compound coenzyme	69.7 ± 8.5	96.3 ± 15.4	68.3 ± 7.6	79.3 ± 11.2	69.2 ± 7.3	65.1 ± 12.3
Sulfur amidine cephalosporin	83.4 ± 7.4	108.3 ± 15.1	84.2 ± 7.6	86.4 ± 13.5	82.8 ± 8.9	70.4 ± 10.5

**Table 2:** Comparison of work efficiency between 1 and 2 dispensing robots and manual dispensing ($\bar{x}\pm s$).

Drug	Operation 1	Artificial blending	Operate two units simultaneously
Card collaterals sulfonic sodium	58.3 ± 8.6	83.4 ± 14.3	92.5 ± 10.3
Cefazoxime sodium for injection	72.4 ± 12.2	91.2 ± 16.1	115.8 ± 9.4
Compound coenzyme	69.7 ± 8.5	96.3 ± 15.4	112.1 ± 7.6
Sulfur amidine cephalosporin	83.4 ± 7.4	108.3 ± 15.1	130.4 ± 9.7

**Table 3:** Comparison of residual amount between dispensing robot and manual.

The serial number	Card collaterals sulfonic sodium		Cefazoxime sodium for injection		Compound coenzyme		Sulfur amidine cephalosporin	
	The robot	Artificial	The robot	Artificial	The robot	Artificial	The robot	Artificial
Dosing of 1	0.123	0.06	0.142	0.125	0.112	0.095	0.145	0.16
Dosing of 2	0.15	0.075	0.147	0.085	0.108	0.055	0.138	0.11
Dosing of 3	0.138	0.05	0.145	0.062	0.122	0.072	0.142	0.11
Dosing of 4	0.128	0.06	0.135	0.102	0.118	0.135	0.14	0.25
Dosing of 5	0.135	0.075	0.133	0.075	0.115	0.085	0.13	0.12
Dosing of 6	0.13	0.03	0.146	0.105	0.126	0.105	0.143	0.17
Dosing of 7	0.138	0.05	0.143	0.09	0.122	0.068	0.145	0.06
Dosing of 8	0.129	0.075	0.122	0.085	0.006	0.076	0.133	0.20
Dosing of 9	0.133	0.095	0.139	0.11	0.112	0.128	0.135	0.08
Dosing of 10	0.128	0.02	0.129	0.12	0.128	0.068	0.144	0.13

Note: the artificial preparation of sodium carlosulfonate, cefazoxime sodium for injection, and complex coenzyme were dissolved with 2-3 mL of solvent; cefathiamidine was dissolved in 5 mL of solvent. The dispensing robot dispenses 2 mL of solvent for compound coenzyme and 3 mL of solvent for sodium carlosulfonate and cefazoxime sodium for injection.

**Table 4:** Comparison of residual quantity between dispensing robot and manual ($\bar{x}\pm s$).

Drug	Residue of dispensing robot	Manually mix residue
Card collaterals sulfonic sodium	0.13 ± 0.01	0.06±0.02^1)^
Cefazoxime sodium for injection	0.14 ± 0.01	0.10±0.02^1)^
Compound coenzyme	0.12 ± 0.01	0.09±0.03^1)^
Sulfur amidine cephalosporin	0.14 ± 0.01	0.14±0.06^1)^

Note: compared with the dispensing robot group, *P* < 0.001.

**Table 5:** Record of manual injection pushing and pulling times.

Drug	Dosing quantity per bag	Times to push and pull the needle
Card collaterals sulfonic sodium	4	834 ± 143
Cefazoxime sodium for injection	2	547 ± 97
Compound coenzyme	2	578 ± 92
Sulfur amidine cephalosporin	2	325 ± 45

Note: the times of pushing and pulling syringes were 10 times per bag of sodium calosulfonate, 6 times per bag of cefazoxime sodium and complex coenzyme, and 3 times per bag of cefathiamidine.

**Table 6:** Comparison of dispensing errors between dispensing robot and manual.

Month	The dispensing robot made a dispensing error	Manual error
1	1	10
2	0	8
3	0	7
4	2	16
5	1	5
6	0	14

Note: there are mainly three types: wrong dispensing, wrong dosage, and no needle change.

## Data Availability

The data used to support the findings of this study are available from the corresponding author upon request.
